# The genome of *Aspergillus niger* strain melanoliber

**DOI:** 10.1128/mra.00809-24

**Published:** 2025-02-18

**Authors:** Pushya Pradeep, Rakshita Sukruth Kolipakala, Deepesh Nagarajan

**Affiliations:** 1 Department of Biotechnology, Faculty of Life and Allied Health Sciences, M. S. Ramaiah University of Applied Sciences, Bengaluru, India; 2 Department of Microbiology, St. Xavier’s College, Mumbai, India; University of California Riverside, Riverside, California, USA

**Keywords:** melanin, mycology

## Abstract

*Aspergillus niger* strain melanoliber was isolated from the coastal region of Mumbai, Maharashtra, India. This strain secretes water-soluble melanin attached to peptides possessing metal-chelation properties. Here, we report the whole-genome sequence of *Aspergillus niger* strain melanoliber. The genome was sequenced using Illumina Novaseq6000 giving a total size of 3.86 Mb.

## ANNOUNCEMENT

*Aspergillus niger* is a fungal species of the taxonomic division Ascomycota with wide-ranging industrial applications, including the production of citric acid and enzymes such as amylase and protease (1, 2). Various fungal species, including *Aspergillus niger*, produce a blackish pigment called melanin (3, 4). A new strain of *Aspergillus niger* has been recently isolated from soil samples in Mumbai, Maharashtra, India—400001. It has been documented that this strain produces a distinct type of melanin that is covalently bound to short peptides (peptidomelanin) and can chelate heavy metals (5). Here, we present the whole-genome sequence of the *Aspergillus niger* strain melanoliber, which may aid in elucidating the enzymes and mechanisms responsible for the production of peptidomelanin.

The *Aspergillus niger* strain melanoliber was grown in Sabouraud dextrose broth/agar at 37°C overnight (5) and the genomic DNA was isolated using the DNeasy PowerSoil Pro Kit (Qiagen Cat. No. 47014) according to the manufacturer’s protocol. The quality of DNA was evaluated using a Qubit 3.0 fluorometer (Thermofisher Scientific, Cat. No. Q33238) using a DNA HS assay kit (Thermofisher Scientific, Cat. No.Q32851) and Nanodrop. The concentration of DNA was 102 ng/µL. The purity ratios 260/280 and 260/230 were 1.83 and 1.87, respectively. The DNA was also analyzed for fragmentation using agarose gel electrophoresis.

The extracted DNA was used to create a DNA library using the Roche KAPA HyperPlus Kit (Cat. No. KR1145 – v8.21), and the library size had an average length of 326 bp. Sequencing was conducted on the Illumina NovaSeq 6000 using the NovaSeq 6000 S4 Reagent Kit v1.5, yielding 37,062,860 reads with an average read length of about 134 bp and GC content of 49.27%. The reads were processed and cleaned through FastP(v0.23.4) (6) to remove adapters and erroneous bases. *De novo* genome assembly of the cleaned reads was performed using the Maryland Super Read Cabog Assembler (MaSuRCA) (v4.1.0) (7) yielding a total size of 48,383,866 bp and GC content of 48.84%. The statistics of the genome assembly is represented in Table 1.

**TABLE 1:** Genome assembly statistics.

Assembly	Sample— *A. niger* melanoliber
No. of contigs (≥0 bp)	1,506
No. of contigs (≥1,000 bp)	1,345
No. of contigs (≥5,000 bp)	1,141
No. of contigs (≥10,000 bp)	983
No. of contigs (≥25,000 bp)	641
No. of contigs (≥50,000 bp)	316
Total length (≥0 bp)	48,408,712
Total length (≥1,000 bp)	48,314,253
Total length (≥5,000 bp)	47,775,399
Total length (≥10,000 bp)	46,616,020
Total length (≥25,000 bp)	40,809,648
Total length (≥50,000 bp)	29,263,557
Total no. of contigs	1,443
Largest contig	383,355
Total length	48,383,866
GC%	48.84
N50	65,210
N90	18,851

**Fig 1:**
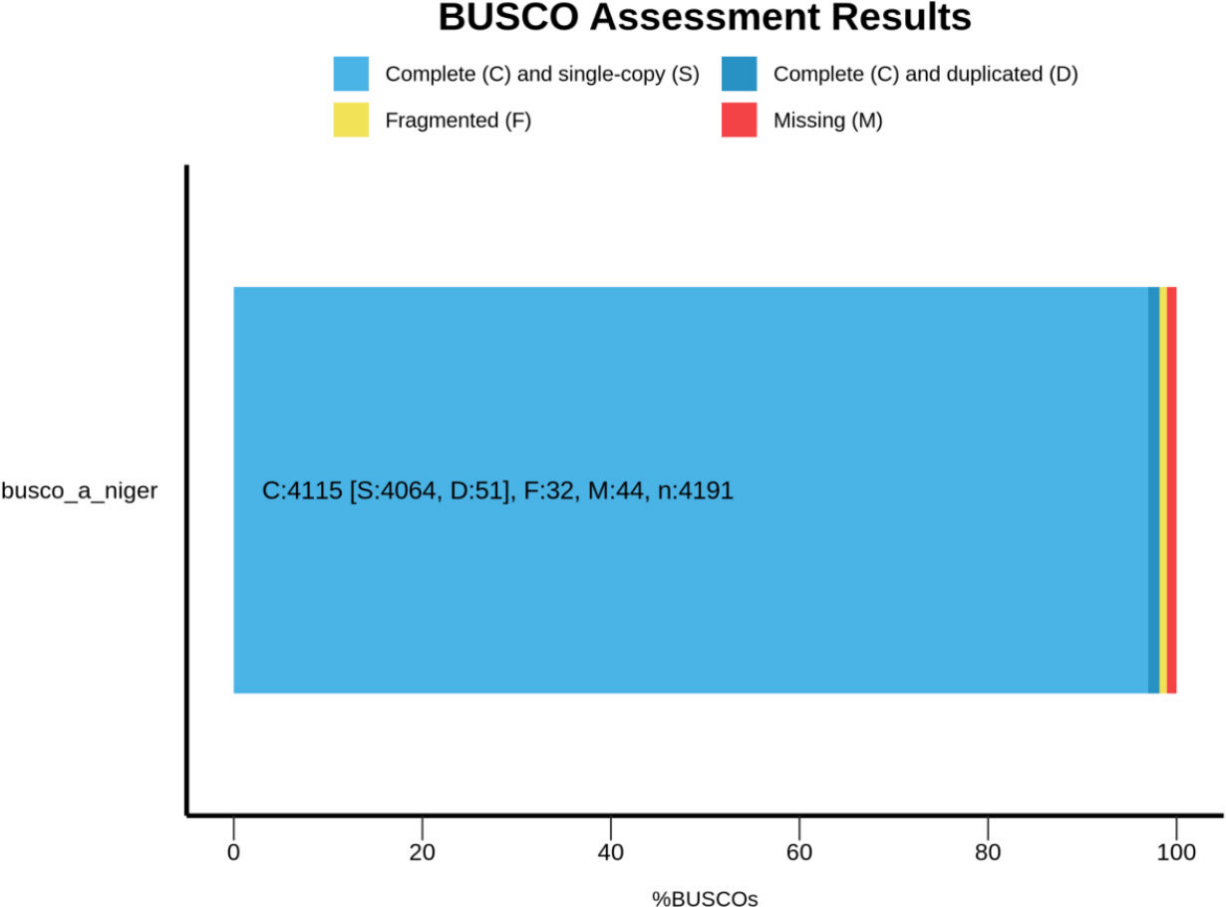
Genome completeness assessment of *Aspergillus niger* strain melanoliber using BUSCO.

The genome was checked for completeness using Benchmarking Universal Single-Copy Orthologs (BUSCO) (v5.7.1) (8), using “fungi_odb1” with completeness being 4,115 out of a total 4,191, single-copy being 4,064, 51 duplicates, and 44 missing genes (Fig. 1).

Gene annotations were completed using GenMark-ES (Eukaryotic Self-training) (v4.72 lic) (9) and EggNOG-mapper (v2.14.1) (10) for gene prediction and functional annotation (11), respectively. Specific parameters such as “fungus” and “target_taxa_ascomycota” were used in GenMark-ES and EggNOG-mapper, respectively, to ensure the prediction of fungal genes. The total number of genes is 18,375, with 44,575 exons and 26,200 introns. In addition, a reference-based annotation was performed using a BLASTp (v2.14.1) (12) search against the *Aspergillus niger* reference genome strain CBS 513.88 (accession no. PRJNA721011) (13). This analysis revealed that 1,784 genes exhibited complete identity, 8,377 genes had over 90% identity, and 5,626 genes showed less than 50% identity compared to the reference genome. This will allow us to further analyze and identify novel genes in *Aspergillus niger* strain melanoliber that may play a role in peptidomelanin formation, utilizing the genome assembly and annotation data.

## Data Availability

The whole-genome sequence has been submitted to GenBank under the number JBECZT000000000.1. The sequence data have been submitted to NCBI-SRA with accession number SRR31522722. The culture strain has been submitted to Microbial Type Culture Collection (MTCC), Chandigarh under strain ID MTCC 13366.

## References

[1] Show PL, Oladele KO, Siew QY, Aziz Zakry FA, Lan JC-W, Ling TC. 2015. Overview of citric acid production from Aspergillus niger . Front Life Sci 8:271–283. doi:10.1080/21553769.2015.1033653

[2] Saranraj P, Stella D. 2013. Fungal amylase—a review. Int. J. Microbiol. Res 4:203–211. doi:10.5829/idosi.ijmr.2013.4.2.75170

[3] Jacobson ES, Tinnell SB. 1993. Antioxidant function of fungal melanin. J Bacteriol 175:7102–7104. doi:10.1128/jb.175.21.7102-7104.1993 8226653 PMC206840

[4] Nosanchuk JD, Casadevall A. 2006. Impact of melanin on microbial virulence and clinical resistance to antimicrobial compounds. Antimicrob Agents Chemother 50:3519–3528. doi:10.1128/AAC.00545-06 17065617 PMC1635213

[5] Schmid JD, Licata A, Goldhardt O, Förstl H, Yakushew I, Otto M, Straub SA, Beer A, Ludolph AC, Landwehrmeyer GB, et al.. 2024. Fungal peptidomelanin: a novel biopolymer for the chelation of heavy metals. ACS Omega 9:36353–36370. doi:10.1021/acsomega.4c03704 39220543 PMC11359623

[6] Chen S, Zhou Y, Chen Y, Gu J. 2018. fastp: an ultra-fast all-in-one fastq preprocessor. Bioinformatics 34:i884–i890. doi:10.1093/bioinformatics/bty560 30423086 PMC6129281

[7] Zimin AV, Marçais G, Puiu D, Roberts M, Salzberg SL, Yorke JA. 2013. The MaSuRCA genome assembler. Bioinformatics 29:2669–2677. doi:10.1093/bioinformatics/btt476 23990416 PMC3799473

[8] Simão FA, Waterhouse RM, Ioannidis P, Kriventseva EV, Zdobnov EM. 2015. BUSCO: assessing genome assembly and annotation completeness with single-copy orthologs. Bioinformatics 31:3210–3212. doi:10.1093/bioinformatics/btv351 26059717

[9] Borodovsky M, Mills R, Besemer J, Lomsadze A. 2003. Prokaryotic gene prediction using genemark and genemark.hmm. CP in Bioinformatics 1:4–5. doi:10.1002/0471250953.bi0405s01 18428700

[10] Diehl-Schmid J, Licata A, Goldhardt O, Förstl H, Yakushew I, Otto M, Anderl-Straub S, Beer A, Ludolph AC, Landwehrmeyer GB, et al.. 2019. FDG-PET underscores the key role of the thalamus in frontotemporal lobar degeneration caused by C9ORF72 mutations. Transl Psychiatry 9. doi:10.1038/s41398-019-0381-1 PMC6355852 30705258

[11] Cantalapiedra CP, Hernández-Plaza A, Letunic I, Bork P, Huerta-Cepas J. 2021. eggNOG-mapper v2: functional annotation, orthology assignments, and domain prediction at the metagenomic scale. Mol Biol Evol 38:5825–5829. doi:10.1093/molbev/msab293 34597405 PMC8662613

[12] Altschul SF, Madden TL, Schäffer AA, Zhang J, Zhang Z, Miller W, Lipman DJ. 1997. Gapped BLAST and PSI-BLAST: a new generation of protein database search programs. Nucleic Acids Res 25:3389–3402. doi:10.1093/nar/25.17.3389 9254694 PMC146917

[13] Pel HJ, de Winde JH, Archer DB, Dyer PS, Hofmann G, Schaap PJ, Turner G, de Vries RP, Albang R, Albermann K, et al.. 2007. Genome sequencing and analysis of the versatile cell factory Aspergillus niger CBS 513.88. Nat Biotechnol 25:221–231. doi:10.1038/nbt1282 17259976

